# Considerations for using potential surrogate endpoints in cancer screening trials

**DOI:** 10.1016/S1470-2045(24)00015-9

**Published:** 2024-05-01

**Authors:** Alexis B Webb, Christine D Berg, Philip E Castle, David Crosby, Ruth Etzioni, Larry G Kessler, Usha Menon, Mahesh Parmar, Robert J C Steele, Peter D Sasieni

**Affiliations:** Cancer Research UK, London, UK; Early Cancer Detection Consultant, Bethesda, MD, USA; Division of Cancer Prevention and Division of Cancer Epidemiology and Genetics, US National Cancer Institute, Rockville, MD, USA; Cancer Research UK, London, UK; Public Health Sciences Division, Fred Hutchinson Cancer Research Center, Seattle, WA, USA; Department of Health Systems and Population Health (HSPOP), School of Public Health, University of Washington, Seattle, WA, USA; Medical Research Council Clinical Trials Unit, Institute of Clinical Trials and Methodology, University College London, London, UK; Medical Research Council Clinical Trials Unit, Institute of Clinical Trials and Methodology, University College London, London, UK; University of Dundee, Department of Surgery, Ninewells Hospital, Dundee, UK; The Cancer Research UK and King’s College London Cancer Prevention Trials Unit, King’s College London, London, UK; Wolfson Institute of Population Health, Queen Mary University of London, London, UK

## Abstract

The requirement of large-scale expensive cancer screening trials spanning decades creates considerable barriers to the development, commercialisation, and implementation of novel screening tests. One way to address these problems is to use surrogate endpoints for the ultimate endpoint of interest, cancer mortality, at an earlier timepoint. This Review aims to highlight the issues underlying the choice and use of surrogate endpoints for cancer screening trials, to propose criteria for when and how we might use such endpoints, and to suggest possible candidates. We present the current landscape and challenges, and discuss lessons and shortcomings from the therapeutic trial setting. It is hugely challenging to validate a surrogate endpoint, even with carefully designed clinical studies. Nevertheless, we consider whether there are candidates that might satisfy the requirements defined by research and regulatory bodies.

## Introduction

Effective cancer screening programmes reduce the number of deaths from cancer.^1,2^ However, evaluating a new test for its likely population benefit is not straightforward. Comparing outcomes of screen-detected cancers with those of symptomatic cancers can be misleading. Randomised controlled trials with cancer-specific mortality reduction as the primary outcome are accepted as the gold standard for evaluation. Such trials need to be large with thousands of participants and a total duration extending from 5 to 15 years. These requirements delay the implementation of effective technologies and encourage continued investment in approaches that might never be effectively deployed in a screening programme.

As new technologies^3–6^ for detecting asymptomatic cancers multiply, there is increased interest in ensuring efficacious tests reach the population in a timely manner. Use of a surrogate endpoint as the primary outcome (replacing disease-specific mortality) has the potential to considerably decrease the time, size, and expense of clinical trials. The term surrogate endpoint is well defined in medical statistics literature, but validation requires multiple randomised controlled trials in which both the true and surrogate endpoints are measured and which are lengthy and expensive (definitions are given in the panel). Once a surrogate endpoint has been validated, this result is applicable only to the conditions of the trial in which it was tested, including the specific screening test used. The concept of potential earlier endpoints remains important; therefore, we consider proxy endpoints. These proxy endpoints might be useful in determining whether screening might be effective in reducing cancer-specific mortality. Adapting George Box’s aphorism: no proxy is a surrogate, but some are useful; by useful we mean that if we conclude that the intervention has a beneficial effect on the proxy endpoint, then we can be confident that it will also have a beneficial effect on the true endpoint. We note that a futility proxy endpoint would be useful when concluding that the intervention has no clinically meaningful effect on the proxy endpoint, then we can be confident that it would have no clinically meaningful effect on the true endpoint. In this Review we use the term surrogate informally except for where it might be confusing when we use the term proxy. Formal definitions of surrogate endpoint and proxy endpoint ane given in the panel.

If a screening test is successful in reducing disease-specific mortality, use of a surrogate endpoint could make the pathway to adoption more efficient; if the test is not effective it would eliminate years of fruitless investment. In practice, no endpoint is likely to have been validated as a surrogate before conducting a randomised controlled trial of screening, which means that there is an inherent risk in drawing the wrong conclusion based on a proxy endpoint. Following a positive result, it is important to consider how best to generate additional data on cancer-specific mortality with extended follow-up or stepped-wedge type implementation studies. This discussion is taking place within the context of development of multi-cancer early detection tests (MCEDs),^7^ which detect and can distinguish molecular profiles of multiple different cancers in biological fluids.

Here, we focus on screening to detect invasive cancers (distinct from treatable precursors) early. We also distinguish between trials to establish efficacy of a new screening test (our focus) and trials of an updated version of a screening test of proven benefit. In the screening for colorectal cancer, faecal immunochemical testing (FIT) has replaced guaiac-based faecal occult blood testing (gFOBt) without direct evidence of FIT’s effects on mortality. The rationale for this change was that (1) randomised controlled trials have shown a reduction in colorectal cancer mortality with gFOBt-based screening,^2^ (2) the pathway from advanced adenomas to colorectal cancer is well documented, (3) trials have shown that participation in FIT-based screening was higher, and (4) the positive predictive value of FIT for both colorectal cancer and for advanced adenomas was higher at the same positivity level than with gFOBt.

**Panel: Definition of key terms**

- Primary outcome: the main measured outcome of a trial that is statistically powered to support a conclusion
- Target endpoint: the clinically important endpoint that is of ultimate interest—typically cancer-specific mortality when evaluating a new cancer-screening test
- Screening test: a test that is used to detect cancer or precancerous conditions in an asymptomatic population
- Surrogate endpoint: a proxy endpoint that mediates the mortality endpoint such that for the intervention to reduce mortality it is both necessary and sufficient that it reduces the proxy
- Proxy endpoint: a cancer-related event measurable in a screening trial that occurs earlier than cause-specific mortality and is used to allow determination of beneficial, harmful, or null effect of the intervention
- Negative surrogate: null effect documented with surrogate that allows early stopping of trial or of individual screening groups for futility
- Efficacy: the effect of an intervention on outcomes under ideal or controlled conditions; usually contrasted with effectiveness, which is the effect of an intervention under average or realistic conditions
- Advanced stage: this has been defined differently according to the cancer and the individual screening trial; definitions have mostly included distant metastatic disease (stages III and IV) with, in some instances, local spread beyond the confines of the organ (stage II+)

This Review considers what it would take to identify an endpoint that can be measured earlier than disease-specific mortality and that could serve as the primary outcome—ie, as a surrogate for disease-specific mortality in cancer-screening trials. We provide a framework that researchers could use when exploring the possibility of using surrogate endpoints. We consider elements of ideal surrogate endpoints with the caveat that they might vary for different cancer–test combinations and that there might not be a perfect surrogate endpoint for the actual outcome of interest, which is disease-specific mortality. A potential surrogate endpoint should only be considered if it would lead to substantial gains by reducing the duration, size, or cost of a clinical trial compared with using disease-specific mortality as an endpoint. Furthermore, we recommend that cancer-specific mortality should continue to be measured as a secondary endpoint in these trials.

### Criteria: applying Prentice and beyond

A surrogate endpoint in a cancer-screening trial is an endpoint that occurs earlier than cancer mortality and is adequate for ascertaining whether the screening confers a clinical benefit. The concept has been thoroughly examined in the setting of cancer treatment trials.^8–10^

Prentice^8^ defined a surrogate endpoint as one that could be used in a treatment trial to test the null hypothesis of the intervention having no effect on the primary outcome; that is, the intervention would affect the primary outcome if and only if it influences the surrogate endpoint outcome, which is a stringent requirement. This stipulation implies that the surrogate endpoint should be a mediator (ie, it should be on the causal pathway between the intervention and the primary outcome) and that there is no direct effect of the intervention on the primary endpoint (ie, all its effects are through the mediator), which is essentially impossible to verify. This definition would also imply that an excellent surrogate endpoint for one intervention might not work at all for another. Empirically, there is a hierarchy of evidence that supports the use of a surrogate endpoint for a particular intervention. The strongest evidence comes from synthesis of randomised controlled trials with meta-regression (via the origin) of the effect of the intervention on the true endpoint against the effect of the intervention on the surrogate endpoint.^11^ However, by the time sufficient evidence is generated, there is little need for a surrogate endpoint.^12^ Next in the hierarchy is data showing that the probability of the true endpoint given the surrogate is independent of the intervention.^13^ Knowledge about the effects of treatment on early cancers and on whether the screen-detectability of the cancer might influence its prognosis can be helpful in deciding whether a surrogate endpoint is reasonable. An understanding of cancer biology in the context of surrogate outcomes is important.^14^

In the setting of cancer-screening trials, the opportunities to find an adequate surrogate endpoint for disease-specific mortality are somewhat limited. Cuzick and colleagues^15^ proposed using stage-based predicted disease-specific mortality. We suggest that a potential surrogate endpoint would have to (1) be measurable sooner than mortality, (2) predict disease-specific mortality measured from the start of the trial, and (3) mediate the effect of screening on disease-specific mortality. Taken together, these factors restrict potential surrogates to endpoints that are related to the temporal course of disease, ruling out prognostic features that are time-invariant.

Rigorously establishing whether an endpoint is an adequate surrogate requires data from multiple trials in which screening had varying effects, and for which information is available on both the putative surrogate and the true endpoint. Even where such data exist, care should be taken if extrapolating use of the surrogate endpoint to a different cancer or screening technology for the same cancer, since a technology might detect a subset of cancers that have a much poorer prognosis (after adjusting for standard prognostic factors) than the subset detected without that technology. Stuart G Baker^11^ proposed five criteria that should be considered for using a surrogate endpoint in a new trial; two are statistical and linked to the results of a meta-analysis, and three are biological.

Use of disease features prognostic for disease-specific survival requires care when used in screening studies. Screening identifies disease earlier than it would otherwise be diagnosed. Thus, changes in the prognostic profile of detected disease might simply reflect a lead-time bias—ie, the cancer is found earlier, but the age at death is unaltered. Disease-specific mortality will be reduced only if screening leads to an improvement in post-lead-time survival, which is survival measured from point of clinical diagnosis in the absence of screening. A candidate surrogate endpoint for screening trials should be strongly associated with mortality (and not just with post-diagnosis survival) from the targeted cancer and that association should remain after adjusting for the route to diagnosis. Maintaining these associations is important to ensure that the cancers diagnosed early with screening can be successfully treated. There is nothing gained by screen-detecting a cancer at an early stage if treatment does not alter its progression to fatality.

### Candidate surrogate endpoints

When discussing surrogate endpoints, it is useful to consider the temporal course of cancer along which proxies could be observed (figure). We discuss separately proxies that could be used to conclude that screening is not effective (ie, early stoppage for futility) and proxies that could be used to conclude that screening is effective. Screening programmes with clinical trial based evidence of cancer-specific mortality reduction include those for lung (low-dose computed tomography [LDCT]),^1,16,17^ breast (mammography),^18^ cervix (human papillomavirus testing),^19^ and colon (endoscopy^20,21^ or FOBt) cancers.^22,23^

Examples of screening clinical trials with no reduction in cause-specific mortality, in which we can examine whether the negative outcome might have been predicted earlier by the surrogate endpoint, include negative trials of screening of proven benefit (listed in the preceding paragraph) and trials of lung (x-ray),^24^ ovary (ultrasound and CA-125 or multimodal screening),^25^ and prostate (prostate-specific antigen [PSA]) cancer.^26^ For prostate cancer, there are some trials showing a reduction in mortality, and others that failed to show such an effect. It would be important to see if the proposed surrogate endpoint captures these differing results and if we could have predicted the outcome of each trial by using the surrogate. Table 1 includes candidate surrogate endpoints for individual cancers, all of which are prognostic factors used clinically to varying extents. These candidates are on the pathway between initiation and death from cancer and have the potential to be affected by screening and early intervention. A key factor determining the feasibility of these candidates relates to whether there exist internationally accepted definitions. The use of a composite outcome across multiple cancers as a proxy for cancer mortality in MCED trials adds further complexity. Considerations for design of MCED trials are discussed in publications by Minasian and colleagues^27^ and Neal and colleagues.^28^

When considering trial design using surrogate endpoints, trialists should continue to consider other aspects of trial conduct. Appropriate inclusion criteria so that trial results can be extrapolated to a broad population (external validity) are important. Also, in cancer-screening trials, it is important to ensure that participants are not too frail to undergo appropriate diagnostic evaluation and therapy for the cancer of interest. Whether these factors are more relevant when using surrogate endpoints when compared with cancer mortality is unknown, but it is possible to contrive hypothetical examples in which poor design choices could lead to a positive conclusion with respect to a surrogate endpoint that would not be reached if based on cancer mortality.

A leading candidate endpoint is advanced-stage disease at presentation. This endpoint captures the rationale underlying early detection, namely, that diagnosing cancers earlier in their natural history allows treatment to be commenced earlier with corresponding extended life expectancy. If the cancer is not diagnosed until it is at an advanced stage, screening (or routine care) has failed in the individual. Use of early-stage disease as an endpoint is not recommended because it is possible to observe an increase in early-stage diagnoses due to over-diagnosis without there being any subsequent reduction in advanced-stage disease or mortality. This problem is seen, for instance, in a trial of breast screening by clinical examination,^29^ and with lung cancer in trials of x-ray screening where more lung cancers were diagnosed in the intervention groups without a decrease in mortality.^30,31^ In the two x-ray-screening trials, there was a reduction in the proportion of lung cancers that were not resectable, but not in the numbers of such cancers. The reduction in advanced-stage diagnosis has long been considered an important prerequisite for mortality benefit, but it might not always be sufficient. Conversely, reduction in tumour volume (facilitating no or minimal residual disease after surgery) even without reduction in advanced-stage disease could contribute to a reduction in mortality.^32^ The precise relationship between reduction in incidence of advanced-stage disease and disease-specific mortality is known for only a few cancers. More crucially, this effect is dependent on the efficacy of treatment for advanced-stage disease and is dynamic, for example survival of patients with advanced-stage prostate cancer has improved markedly in the last 5 years and the use of maintenance therapy in ovarian cancer is improving survival of advanced disease in the decade following the UK Collaborative Trial of Ovarian Cancer Screening (UKCTOCS).^33,34^

When considering a feature of the cancer (eg, metastases) as a surrogate endpoint, it is desirable to use a cumulative measure. Rather than only using metastases at diagnosis (ie, advanced stage), metastases that develop during follow-up of early-stage disease should be included. A trial with this endpoint would have the same duration as one looking at advanced stage but would re-categorise an individual whose cancer progressed during that initial phase of follow-up as having a poor outcome. An advantage of this endpoint is that it closely tracks with subsequent cancer-specific mortality. However, a drawback of this endpoint is that ascertaining metastatic progression can be challenging without close prospective follow-up and is highly dependent on the intensity of post-diagnosis surveillance. Staging at diagnosis on the other hand has the benefit of codification with the American Joint Committee on Cancer or Union for International Cancer Control approaches that have been refined over decades even though staging might be dependent upon the technologies available and is not a precise science.

Particularly for cancers for which late-stage disease at diagnosis is often not fatal, absolute reduction in advanced stage might be considered a primary endpoint,^35^ because both the side-effects and the cost of treatment are often considerably greater for advanced-stage disease.

Another approach is to use mortality predicted by a stage-shift model as the surrogate endpoint.^12,13^ In practice, this approach would amount to predicting the mortality by a specific time following randomisation (eg, 10 years) given the stage and time of cancer diagnoses in the experimental and control groups at an earlier time (eg, 3 years from last randomisation). Predicting mortality at a given time post-randomisation should address problems of lead-time bias. Predicted mortality will be conservative in the presence of over-diagnosis, or with insufficient follow-up, resulting in greater cancer incidence in the screening group at analysis. Conversely, predicted mortality could be used for screening tests that lead to both early detection of invasive cancer and pre-cancerous lesions, and subsequent prevention of cancer. If stage-specific survival differs between screen-detected cancers and symptomatic cancers, then the method would be biased (in either direction).

Early-stage cancers detected using circulating tumour DNA (ctDNA) tests might have worse prognosis than clinically diagnosed early-stage cancers,^36^ but this has only been shown by testing blood samples post-diagnosis. A seemingly localised cancer that is detectable by a ctDNA test might be further advanced than localised cancers diagnosed following symptoms or other types of screening. For example, some biomarkers for colorectal^37^ and lung cancers^38,39^ are only released from cancers with poor prognosis. In such cases, early-stage screen-detected tumours could already be on a trajectory to lethality and early detection might not alter the outcome. The surrogate endpoint would need to also take this problem into account and predict outcomes dependent on this molecular phenotype.

Increasingly, molecular characterisation provides new prognostic information beyond traditional factors such as tumour stage.^40^ However, it is unclear whether screening can alter the incidence of adverse molecular profiles. Apart from prognostic biomarkers established at diagnosis, there are others that could be assessed later along the cancer pathway. For instance, cumulative incidence of more than minimal residual disease at the end of first-line treatment or of relapse at pre-specified timepoints from randomisation could be used. The advantages and disadvantages of these endpoints are detailed in table 1.

### Evidence for advanced-stage or stage-based predicted mortality as a surrogate endpoint

Meta-regression of the relative effect on the surrogate endpoint versus the relative effect on the true endpoint (constrained so that the regression line runs through to origin corresponding to no effect on either the surrogate or the true endpoint) is the most appropriate method for assessing surrogacy.^9–13^ However, we note that a perfect surrogate might have a slope that is far from 1·0 and depending on the size of the trials in the analysis, there is no reason to expect the R-squared value to be close to 1·0. A slope of 1·0 would indicate that the effect of screening on the surrogate is quantitatively similar to its effect on mortality. An R-squared value measures the degree of correlation between the two variables in the regression. A value of 1·0 would indicate that the data lie exactly in a straight line. Since in a clinical trial both the surrogate and cancer mortality are subject to random variation, we would not expect all datapoints to line exactly on the best fitting straight line. The magnitude of R-squared depends both on the variance of the estimated true effect in each trial and the variance of the true effect across trials. The variance of the true effect for individual trials depends on the size of the trials (and the duration of follow-up) while the variance of true effect across trials depends on the homogeneity of the interventions across the trials. A detailed discussion of statistical methodology for validating a potential surrogate endpoint is beyond the scope of this Review.

Autier and colleagues^41^ showed a positive correlation between advanced-stage (stage III and IV) incidence reduction and breast-cancer mortality reduction across most mammography-screening trials with just one outlying result. Similarly, Tabár and colleagues^42^ found a direct correlation between breast-cancer mortality and incidence of advanced stage cancers. When Duffy and colleagues^43^ defined advanced disease as “invasive breast cancer measuring >20 mm and/or with ≥4 metastatic axillary lymph nodes”, they too found a correlation at the county level (weighted analysis of the results presented in figures 2 and 4 of their paper)^43^ between the reduction via mammography in (1) the number of cases of advanced disease and (2) breast cancer-related mortality. However, the effect of screening on breast-cancer mortality was greater than its effect on advanced-stage breast cancer (41% reduction *vs* 24% reduction), noting that sometimes the relative effect on the surrogate endpoint will be greater than on the target endpoint and sometimes less. Modelling and meta-analysis should be used to calibrate the magnitude of the intervention on cancer-specific mortality with the magnitude of its effect on the surrogate endpoint.

Owens and colleagues^44^ developed a mathematical formula for predicted mortality reduction given by the stage-shift model (with exponential distributions for cancer incidence and mortality) and assuming stage-specific mortality is independent of the route to diagnosis. They explored whether this prediction matched the results of the trials of prostate, lung, and ovarian cancer screening. In the European Randomized study of Screening for Prostate Cancer trial of prostate cancer screening, the cumulative incidence of advanced-stage cancer (metastatic disease or measured PSA more than 100 ng/mL) was reduced by 48% in the screened group. Owens and colleagues found that the observed 21% (95% CI 9–32%) reduction in prostate cancer mortality almost exactly matched the predicted reduction based on the stage-shift model.^44^ They did not examine the Prostate, Lung, Colorectal and Ovarian Cancer Screening trial in which there was no stage-shift and no mortality reduction for prostate cancer. A possible explanation for the lack of effect could be contamination as over 80% of participants in the control group received a PSA test.^45^

Both the National Lung Screening Trial (NLST)^16^ and Nederlands–Leuvens Longkanker Screenings Onderzoek (NELSON)^1^ trial found a reduction in both advanced-stage (III and IV) cancer incidence, and in lung cancer mortality in patients randomly assigned to LDCT screening. In NLST, a decline in advanced-stage disease incidence appeared in the second round of screening. The cumulative incidence was reduced by 21%, which was predictive of the observed mortality reduction of 20% (95% CI 7–27%),^16^ but greater than the modelled mortality reduction of 10%.^44^ UKCTOCS showed a 25% (95% CI 2–42%) decrease in stage IV incidence and a 10% decrease (95% CI 2% increase to 21% decrease) in stage III and IV disease.^25^ The decrease in advanced stage disease occurred in the aggressive high-grade serous cancer subtype and was accompanied by improvements in treatment outcomes.^46^ UKCTOCS showed a 4% decrease in mortality (95% CI 10% increase to 17% decrease). Although the stage IV incidence reduction was statistically significant whereas the mortality reduction was not, the similarity of the confidence intervals for stage III and IV disease and for mortality means that the trial provides little evidence for or against the suitability of advanced stage diseases as a surrogate endpoint for mortality.

In summary, the evidence to date suggests strongly that reduction in advanced-stage presentation due to screening is predictive of a mortality benefit for some cancers, but the quantitative relationship between advanced-stage detection reduction and mortality reduction is cancer specific and depends on the definition of advanced-stage. A surrogate endpoint for one cancer might not be extrapolatable to another cancer and is also potentially screen-technology specific—it might, for instance, hold true for imaging, but not for a blood-based biomarker. Furthermore, the standard stage-shift model ignores prognostic subtypes. It is yet to be established whether mortality predictions from a stage-shift model that preserves prognostic subtypes could be more accurate.

### Regulatory and reimbursement experience with surrogate endpoints

The regulatory landscape for cancer screening will present challenges for proponents of surrogate endpoints, although there are examples of the use of these endpoints in market authorisation for some screening tests. Screening technologies, including assays for biomarker assessment, are classified as medical devices. The regulatory requirements to obtain a screening indication and reimbursement are different in the UK, the EU, and the USA. In the USA, for a medical device to obtain a screening claim, evidence of the possible clinical effect is required by the US Food and Drug Administration (FDA) Center for Devices and Radiological Health. The intended use of the diagnostic test is an intrinsic part of the medical device under USA regulatory schemes. One example of a screening technology accepted by the FDA is Cologuard, which was approved in 2014.^47^ Specifically, “Cologuard is indicated to screen adults of either sex, 50 years or older, who are at typical average-risk for CRC [colorectal cancer]”.^47^ Because there was a well recognised, clinically accepted, and validated technology for screening for colorectal cancer, the sponsor was able to perform a pivotal prospective study on measures of accuracy of the device relative to FDA standards, but did not need to show a reduction in mortality. In table 2, we show generic categories of cancer-screening tests, their use, and whether specific devices in these classes were cleared or approved by the FDA on the basis of a mortality or surrogate endpoint. In 1994, the FDA approved the PSA test in conjunction with a digital rectal examination, as a method for detection of prostate cancer (table 2).^48,49^

Even with FDA clearance or approval for a diagnostic test, there are other hurdles in the USA, chiefly in the form of reimbursement, which is based on effectiveness or clinical utility. Several paths to reimbursement are possible, including acceptance by the United States Preventive Services Task Force with an A or B grade.^50^ An additional pathway to market combining the FDA and Center for Medicare and Medicaid reimbursement hurdles is called coverage with evidence development.^51^ This process provides a pathway while awaiting definitive study evidence, which could include interim use of surrogate endpoints and later confirmation of their predictive ability regarding ultimate endpoints.

At the time of device approval, American regulators might require post-market surveillance as part of the ongoing monitoring of the device. In the case of Cologuard, for example, the FDA required a monitoring study as a post-approval condition. This monitoring study, still in progress, has potential utility in the evaluation of any screening technology that used surrogates as primary endpoints in its definitive trials.

In the UK, screening technologies are regulated under the Medicines and Healthcare products Regulatory Agency (MHRA). The MHRA is currently regulated under the EU regulations for in vitro diagnostic devices and the MHRA require European Commission marking. Screening programmes are only introduced after a recommendation from the UK National Screening Committee. They state that “There should be evidence from high quality randomised controlled trials that the screening programme is effective in reducing mortality or morbidity”, and that the “opportunity cost of the screening programme… should be economically balanced in relation to expenditure on medical care as a whole”.^52^ Cost-effectiveness in cancer screening is generally assessed with health economic modelling and inevitably relies on some extrapolation. In such a context, using a surrogate endpoint requires some assumptions connecting it to patient outcomes (eg, mortality and hence quality-adjusted life-years). It is complex, and often challenging, but routine to estimate cost-effectiveness from trials and to include a probabilistic sensitivity interval on the effect of the uncertainty of the relationship between the surrogate endpoint and outcomes important for cost-effectiveness.

In summary, the absence of published guidelines or standards for the use of surrogate endpoints for the UK, EU, or USA regulatory processes suggests that efforts to reach a scientific consensus of such criteria would be advantageous to the development of screening tests, as it would provide assurances of the requirements to gain market access. Such assurances tend to incentivise technology investment.

### Rapid technology development brings challenges to existing paradigms

Currently, there are considerable barriers to the development, commercialisation, and implementation of cancer screening approaches due to high costs and lengthy timelines. The role of surrogate endpoints, if validated, could have an effect in expediting this process. For emerging single cancer tests where mortality benefit from screening has already been shown with an established (or predicate) test, recommending implementation of a new test based on a previously validated surrogate endpoint could be acceptable. However, for cancer sites with no established screening, including MCEDs, the picture is much more complex. GRAIL’s Galleri test is being evaluated in a large randomised controlled trial in 140 000 asymptomatic participants within the UK’s National Health Service (NHS).^28^ The Galleri test measures methylation of cell-free DNA in over 100 000 DNA regions and uses an artificial intelligence algorithm to identify methylation patterns associated with cancer. The primary outcome is incidence of advanced-stage disease (stage III–IV) 36 months after the last participant is randomly assigned. Trial participants will be followed up for 6 years after the last participant is randomly assigned to see whether there is a cancer mortality benefit. Supporters of this study believe that it is appropriate to plan for widespread implementation of MCED population screening based on a reduction in advanced-stage disease while generating further data on cancer-specific mortality.^28^

If successful, NHS England are planning “to roll out the test to a further one million people across 2024 and 2025.”^53^ Furthermore, assuming the primary outcome results are in line with expectations, the NHS plan “a population screening program from early 2026”.^54^ Details of what constitutes as successful or in line with expectations are not in the public domain. Results from this trial and the decision-making process in the UK will no doubt be studied closely.

By contrast to this accelerated approach, the US National Cancer Institute have announced a 4-year Vanguard study evaluating multiple MCED tests compared with the current standard of care, commencing in 2024.^55,56^ The study will inform the National Cancer Institute on the design of a definitive randomised control trial to determine whether MCED screening reduces cancer mortality.

The demand for expedited surrogate endpoint-driven processes will increase as the pace of technology change accelerates. The screening technologies for breast (transition from analogue to digital x-ray) or lung cancer screening (LDCT) have remained static over the period during which those screening approaches were evaluated, hence the technologies remained relevant by the time of approval and implementation. However, the liquid biopsy field is evolving rapidly. It is possible that such technologies used in a 10-year mortality-powered screening trial would be obsolete by trial’s end. Furthermore, biomarker signatures are being derived by artificial intelligence or machine learning, which is advancing even more rapidly. For these reasons, the case to reimagine how we evaluate cancer-screening tests is more pressing.

### Looking ahead

One route to build the evidence base around surrogate endpoints is to systematically evaluate data that already exist.^57^ Cancer Research UK recently called for proposals to review the literature on cancer-screening trials, across all technologies and cancer types, and to systematically assess whether there is any evidence of surrogacy—that is, of indices measured during the conduct of trials that predicted the ultimate clinical outcomes.^58^ A systematic effort of this nature might help to solidify or refute the case for candidate surrogate endpoints.

**Search strategy and selection criteria**

Search strategy and selection criteria were established by the author group via a series of conference calls that identified the following concepts as relevant for the Review: “primary endpoints or outcomes”, “surrogate endpoints or outcomes”, “cancer-specific mortality”, “cancer screening trials”, “screening test”, “multi-cancer detection tests”, “surrogate endpoints in therapeutic trials”, and “cancer stage (early stage, late stage)”. Individuals or pairs of authors were assigned the task of drafting of different sections of the Review. Each author conducted individual searches via PubMed during the period of drafting from June, 2022 to June, 2023, or of their own files in drafting their contributions to the manuscript. Exact search terms included, but were not limited to, the phrases “primary endpoints or outcomes”, “surrogate endpoints or outcomes”, “cancer-specific mortality”, “cancer screening trials”, “screening test”, “multi-cancer detection tests”, “surrogate endpoints in therapeutic trials”, and “cancer stage” (early and late stage). Only papers published in English were reviewed. All authors read and commented on all sections and could suggest additional publications. The final reference list was generated on the basis of originality and relevance to the broad scope of this Review.

Another approach is to use core outcome sets^59^ for screening trials. If after such a review hypothetical candidate surrogates A, B, and C are of interest, then all future cancer-screening trials might be expected to include measurement and reporting of A, B, and C. Funders could set the expectation that these core outcomes are included in trial design.

Crucial to future endeavours in this space is to ensure appropriate patient and public involvement and education. If screening is introduced based on a surrogate outcome, it is important that the public understand the implications of the results. Communication of the uncertainty due to the imperfect nature of any underlying health states and prediction of future health trajectory is an important consideration. The public should be involved in considering the implications for the delay in roll-out of a new screening technology if surrogate endpoints are rejected, and also the implications of rolling out an ineffective screening test following adoption on the basis of an unproven surrogate endpoint.

Last, researchers considering screening trial surrogate outcomes should be mindful of the pathway towards regulatory approval. If a surrogate was to be used in a trial, what additional evidence would be required before the public were offered screening using the new technology and what post-marketing surveillance should be mandated.

A surrogate endpoint should, on average, give the same qualitative answer as using the true endpoint in an appropriately powered trial. Although surrogates can be found for specific types of screening, it is unlikely that a generic perfect surrogate exists. Each type of cancer and its natural history differs. Nevertheless, by considering how screening works, there is scope for intermediate outcomes that can be used as partial surrogate endpoints for particular purposes.

Since most cancers progress in stages and death from cancer is more common with more advanced stages, it is natural to consider surrogates on the basis of a change in stage distribution. Looking at the proportion of cancers that are of advanced stage is subject to bias from both over-diagnosis and lead time. Rather, a surrogate endpoint should be selected on the basis of the rate of advanced cancer incidence or the predicted number of deaths from the cancers diagnosed in each group in a trial. These measures will fail if the subset of cancers found by screening are at an early stage, but nevertheless prove fatal. Despite the challenges, there is great potential for the sensible use of surrogates to accelerate the translational pathway by stopping trials of ineffective screening technologies early and by progressing implementation research (or even pilot programmes) of promising screening strategies while awaiting cancer mortality results. Further research is needed to identify these endpoints.

## Figures and Tables

**Figure:**
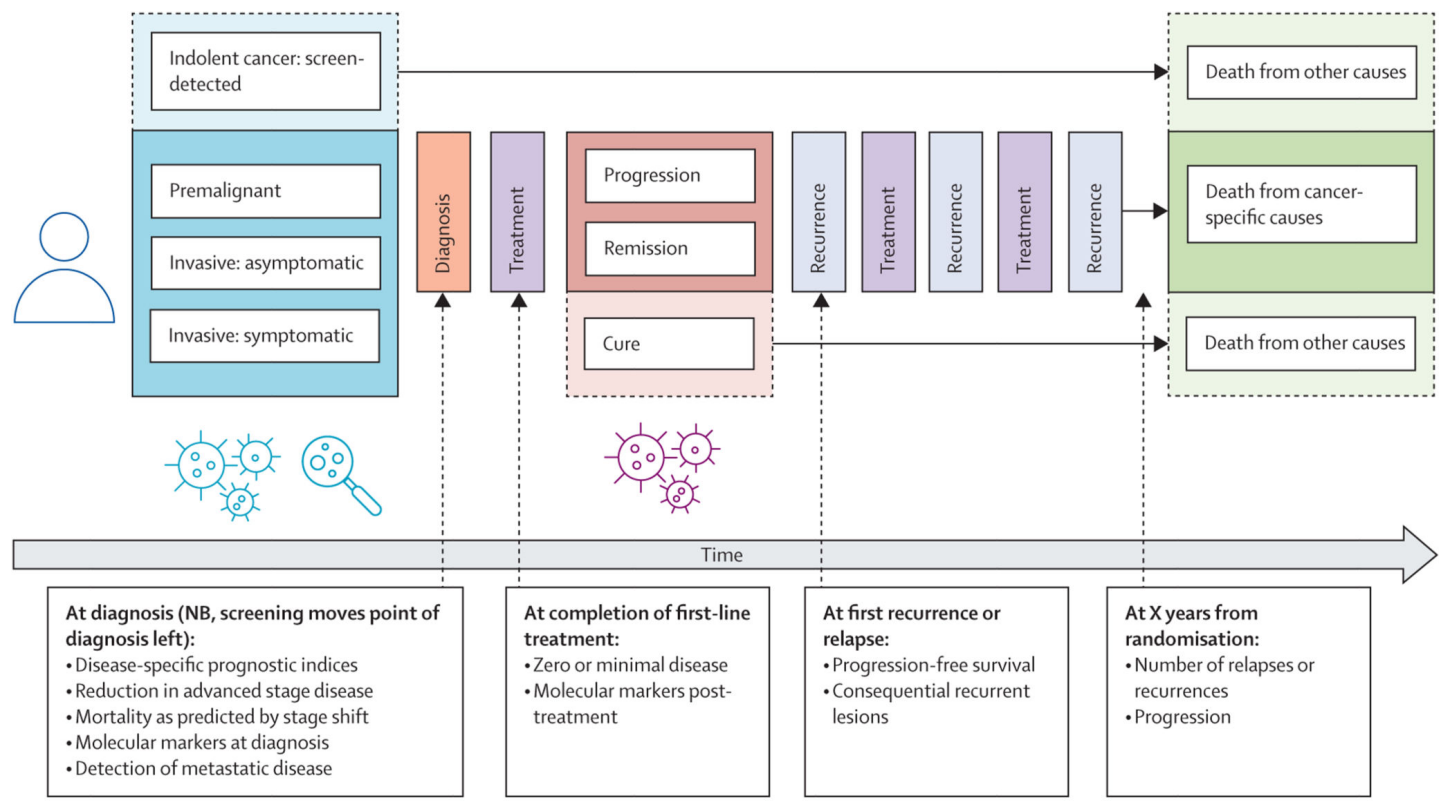
Timing of potential surrogate endpoint candidates during natural history of cancer Dotted lines link boxes with potential surrogate endpoints that could be measured at the time of an event to the box denoting occurrence of the event in the progression diagram. Dotted lines around boxes indicate points outside of cancer-related disease (eg, indolent cancers, curative treatment, and death from other causes). NB=Nota bene. X=number.

**Table 1:** Potential candidates as interim endpoints for mortality to be used in combination with disease-specific mortality in screening trials

	Ascertainment	Advantages	Disadvantages
**Candidates at diagnosis provide considerable time advantage to outcome ascertainment, but are subject to lead time bias**			
Reduction in advanced stage disease incidence	Internationally agreed criteria for staging routinely done with no additional cost for ascertainment	Accepted correlation between stage and disease-specific survival in symptomatic patients; and evidence from screening trials of a correlation with mortality reduction in breast, lung, and possibly colon cancer	Relationship between reduction in incidence of advanced stage disease and disease-specific survival is unknown for most cancers, especially the effect size; the relationship is dependent on the effectiveness of treatment for advanced stage and therefore is dynamic; molecular characterisation of cancers has increasingly shown that poor prognosis is not entirely captured by staging; accurate staging is not a precise science, and often dependent on technologies that are not or cannot be uniformly applied; and the definition of advanced stage disease varies between cancers
Reduction in metastatic disease	No agreed definition, could include locally metastatic disease (stage II-IV) or only distant metastasis (stage IV)	Distant metastasis might have a closer relationship to disease-specific survival; similar advantages as the reduction in advance stage disease incidence apply; and this would include early-stage cancers that recur during follow-up	Similar disadvantages as the reduction in advanced stage disease incidence apply
Reduction in cancers with adverse molecular profile or poor prognostic scores on disease-specific prognostic indices	All need standardisation, different technologies for ascertainment depending on molecular marker; and molecular indices might be missing from many cancers in a large screening trial and more likely to be missing in the control group	Increasingly providing more accurate or new prognostic information beyond traditional factors such as cancer histology; and with newer targeted therapies, might provide better correlation with survival	Few markers currently available; restricted evidence often from small datasets; being tested in treatment trials for stratification, but not yet developed to the point that they can be widely used; and no evidence from screening trials that aggressive cancers detected earlier have a different molecular profile; and the prognostic index might not be on a causal pathway that can be altered by screening, for instance the index might be based on a feature that does not evolve as the cancer progresses (an example that would be inappropriate is human papillomavirus positivity in oropharyngeal cancers)
Predicted mortality from stage at (and date of) diagnosis	Apply stage-specific excess hazards of dying to cancers diagnosed in the trial to predict cancer deaths in each arm at some future date	More nuanced than simply looking at advanced stage; better captures contributions of cancer diagnosed at different stages to overall cancer mortality; and will be more conservative than the presence of over-diagnosis	Relies on independence of stage-specific survival and route of diagnosis; and potential biases from under-staging of screen-detected (asymptomatic) cancers, over-diagnosis, length bias, and screening assay detecting a hallmark of aggressiveness
**Candidates at completion of first-line treatment**			
Decrease in incidence of patients with residual disease	Using a blood test for biomarkers, such as ctDNA, oncoproteins, or imaging alone or in combination	Increasing evidence that ctDNA levels are an indicator of poor prognosis and correlate more accurately with disease progression than stage or non-specific markers	Numerous technical issues still need to be resolved with regard to ctDNA assays; and assays are being tested for monitoring in treatment trials, but data on correlation with mortality are still restricted
**Candidates after first-line treatment (during follow-up)**			
Decrease in incidence of patients with progression, active disease, or relapse	Using imaging or RECIST criteria, but increasingly using blood ctDNA	There is clear evidence from randomised controlled trials of the correlation between progression-free survival (lack of recurrence) and overall survival	With no macroscopic disease, easy to define progression; more difficult if residual disease on initial imaging following the end of first-line treatment; and the need to change standard follow-up in trial to incorporate additional scans and blood tests

ctDNA=circulating tumour DNA. RECIST=Response Evaluation Criteria In Solid Tumours.

**Table 2:** Summary of devices cleared or approved by the FDA with a cancer screening indication and the endpoint used

	Screening indication	Endpoint
Pap smear	Cervical cancer	Detection of cervical cancer or CIN*
HPV screening	ASC-US	Detection of ASC-US†
Mammography	Breast cancer	Breast-cancer mortality
Prostate-specific antigen	Prostate cancer	Prostate cancer detectionf
Low dose CT	Lung cancer	Lung-cancer mortality
Faecal occult blood test	Colorectal cancer	Colorectal-cancer mortality
Faecal immunochemical test	Colorectal cancer	Detection of colorectal cancer†
Cologuard	Colorectal cancer	Detection of colorectal cancer†

ASC-US=atypical squamous cells of undetermined significance. CIN=cervical intraepithelial neoplasia. FDA=Food and Drug Administration. HPV=human papillomavirus.

* The original uses of the Pap test in the 1950s pre-dated any regulation by the FDA (at that time, the FDA regulated all medical products as drugs). Subsequent Pap-related tests have been approved or cleared, but none with a mortality endpoint.

† Surrogate endpoint.
